# Integration of Meta-Multi-Omics Data Using Probabilistic Graphs and External Knowledge

**DOI:** 10.3390/cells12151998

**Published:** 2023-08-04

**Authors:** Handan Can, Sree K. Chanumolu, Barbara D. Nielsen, Sophie Alvarez, Michael J. Naldrett, Gülhan Ünlü, Hasan H. Otu

**Affiliations:** 1 Department of Electrical and Computer Engineering, University of Nebraska-Lincoln, Lincoln, NE 68588, USA; 2 Department of Animal, Veterinary and Food Sciences, University of Idaho, Moscow, ID 83844, USA; 3 Proteomics and Metabolomics Facility, Nebraska Center for Biotechnology, University of Nebraska-Lincoln, Lincoln, NE 68588, USA; 4 Department of Chemical and Biological Engineering, University of Idaho, Moscow, ID 83844, USA; 5 School of Food Science, Washington State University, Pullman, WA 99164, USA

**Keywords:** multi-omics, kefir, *Lentilactobacillus kefiri*, *Lactobacillus kefiranofaciens*, Bayesian networks

## Abstract

Multi-omics has the promise to provide a detailed molecular picture of biological systems. Although obtaining multi-omics data is relatively easy, methods that analyze such data have been lagging. In this paper, we present an algorithm that uses probabilistic graph representations and external knowledge to perform optimal structure learning and deduce a multifarious interaction network for multi-omics data from a bacterial community. Kefir grain, a microbial community that ferments milk and creates kefir, represents a self-renewing, stable, natural microbial community. Kefir has been shown to have a wide range of health benefits. We obtained a controlled bacterial community using the two most abundant and well-studied species in kefir grains: *Lentilactobacillus kefiri* and *Lactobacillus kefiranofaciens*. We applied growth temperatures of 30 °C and 37 °C and obtained transcriptomic, metabolomic, and proteomic data for the same 20 samples (10 samples per temperature). We obtained a multi-omics interaction network, which generated insights that would not have been possible with single-omics analysis. We identified interactions among transcripts, proteins, and metabolites, suggesting active toxin/antitoxin systems. We also observed multifarious interactions that involved the shikimate pathway. These observations helped explain bacterial adaptation to different stress conditions, co-aggregation, and increased activation of *L. kefiranofaciens* at 37 °C.

## 1. Introduction

Individual omics data, such as transcriptomics, proteomics, or metabolomics, can be reliably obtained for a biological system, and analyzing each omics data type separately provides its own unique insight. However, biological systems operate through interactions between different omics types [1]; hence, there is a need for methods that integrate and analyze multi-omics data to uncover the complex and emergent relationships in biological systems [2].

There are various methods that attempt to integrate and analyze multi-omics data [3]. At the integration level, “data-based” approaches concatenate different omics datasets [4], and “model-based” approaches combine individual models (or results) for each omics dataset. At the analysis level, “dimension reduction” methods discover latent features combining different omics and characterize samples [5,6], “correlation, association, or clustering” methods try to elucidate similarity across heterogeneous omics types via group membership [7,8,9,10], and “network” methods attempt to identify the interaction structure between heterogeneous omics types [11,12,13,14]. However, network methods can be limited to certain omics types, e.g., copy number variation and gene expression [11], or target applications other than building interaction networks, e.g., disease subtyping [13].

We previously developed a tool called OBaNK, which is a hybrid data/model-based multi-omics integration method that generates multifarious interaction networks [15]. OBaNK applied Bayesian networks (BNs) to the integration of multi-omics data using external knowledge. By “external knowledge,” we mean molecular interaction evidence and models derived from publicly available sources. Existing biological knowledge is currently stored in heterogeneous forms across multiscale databases. Constructs such as OBaNK that benefit from this wealth of information in a structured way show improved performance [16]. BNs are viable tools for exploring omics data in a graph setting as biological networks consist of linear and nonlinear interactions, which are stochastic, obscured by noise, and can be related to causal inference [17]. BNs reflect the dependency structure for a set of random variables and can be used to precisely capture these kinds of interactions, justifying their use in biological network analysis.

However, OBaNK was limited to human data only and provided a suboptimal integration of external knowledge as it used a maximum likelihood model instead of a maximum *a posteriori* one. We had also developed a construct called Bayesian network prior (BNP), which optimally integrated external knowledge to identify interaction networks using a maximum *a posteriori* model [18]. Unfortunately, BNP is limited to human single-omics data and cannot handle multi-omics data. In this paper, we improve on both previous approaches in two different facets. We develop a method that generates interaction networks for multi-omics data from a community (as opposed to single omics from a single organism) while optimizing the integration of external knowledge using a maximum *a posteriori* model. The proposed method uses multi-omics data from a bacterial community, which we refer to as “meta-multi-omics data.” We use the prefix “meta” like it is used in the term “metagenomics” to denote that the corresponding omics data types are coming from a community of organisms instead of a single organism.

Microbial communities are complex systems that require advanced computational tools to characterize their diversity, stability, and dynamics. Kefir grain, a microbial community that ferments milk, represents a self-renewing, stable, natural microbial community [19]. We and others have investigated the bacterial composition of kefir grains and their biochemical properties [20,21,22,23]. Kefir is a traditional dairy product and a very popular probiotic obtained via the fermentation of milk by kefir grains [24]. Kefir has been shown to be associated with a wide range of health benefits, such as enhancing the immune system, providing anti-inflammatory effects, promoting digestive health, preventing cardiometabolic diseases, and regulating plasma glucose levels, in addition to its antimicrobial and antioxidant capacities [21,23,25,26].

Understanding the structure and stability of the microbial community in kefir grains is important for the success of production strategies and the use of kefir as a probiotic. However, developing a multi-omics interaction network for the complete grain is an extremely difficult and complex task. Therefore, to use it as a testbed for algorithm development, we initially obtained a controlled bacterial community using the two most abundant and well-studied species in kefir grains: *Lentilactobacillus kefiri* and *Lactobacillus kefiranofaciens*. Our overall goal was to establish the molecular dynamics in the controlled bacterial community when we contrasted the growth conditions at 30 °C and 37 °C, the latter aiming to approximate the human body temperature. We obtained transcriptomic, metabolomic, and proteomic data for the *same* 10 biological replicates at each temperature (3 omics data types for 20 samples).

We analyzed individual omics data by identifying statistically significantly differentially expressed molecules between the two temperature settings, assessed the clustering and classification performance of these molecules, and obtained their functional characteristics at the system level. Then, we generated an interaction network that involves all three omics data types using BNs and the incorporation of external knowledge. We compared our results to those of OmicsNet [4], a multiomics integrator using external knowledge, and a correlation-based network-generating approach [10].

Discovering the network of interactions between different types of omics data has the potential to lead to a deeper understanding of the underlying, generalizable biological mechanisms. The method proposed in this paper provides such discoveries with the ability to (i) use multi-omics data from a microbial community as opposed to a single organism, (ii) incorporate far-reaching external knowledge about molecular interactions, (iii) use this external knowledge in its model instead of using it for simple soft/hard decision-making on the existence of interactions, and (iv) optimize the true objective function, MAP, instead of the suboptimal ML. The algorithm developed in this paper, called MEta-Multi-omics Integration using Networks and EXternal knowledge (MEMINEX), is freely available as a software package at http://otulab.unl.edu/MEMINEX (accessed on 1 August 2023).

## 2. Materials and Methods

### 2.1. Strains, Media, and Growth Conditions

We obtained *L. kefiri* (JCM5818) and *L. kefiranofaciens* subsp. *kefiranofaciens* (JCM6985) from the Japan Collection of Microorganisms (JCM). Both *L. kefiri* and *L. kefiranofaciens* were separately activated on Lactobacillus MRS (Difco, Becton, Dickenson, and Co., Sparks, MD, USA) agar plates and incubated anaerobically for 3 days at 30 °C. A single colony from each microorganism was picked, transferred into 15 mL MRS broth, and incubated anaerobically at 30 °C for 3 days [27]. The two strains of bacteria were subcultured twice until they reached the exponential growth phase. In Supplementary Figure S1, we show growth curves for the two bacteria and their co-culture at both 30 °C and 37 °C.

### 2.2. Sample Collection

All samples were initially inoculated with a mixed culture of *L. kefiri* and *L. kefiranofaciens* inoculum blended from 3-day cultures. An amount of 20 mL of MRS medium was inoculated with 0.5 mL of the combined inoculum and incubated for 3 days, one set of 10 at 30 °C and another set of 10 at 37 °C. For controls, *L. kefiri* and *L. kefiranofaciens* were inoculated in triplicate for each temperature. The tubes were removed from the incubator after 3 days, and 0.1 mL of each sample was placed in a 96-well plate for OD (600 nm) analysis. Samples were then centrifuged (3000× *g*, 4 °C, 10 min), and the supernatant was removed and frozen for further analysis. The pellet was washed in fresh MRS (pH 5.5), divided into three aliquots, re-pelleted in microcentrifuge tubes, and frozen at −80 °C for subsequent transcriptomic, proteomic, and metabolomic analysis. All omics experiments and subsequent differential expression analysis were performed on co-cultures.

### 2.3. Transcriptomics

RNA was isolated using Omega Biotek E.Z.N.A.^®^ Total RNA Kit II (Omega Biotek, Norcross, GA, USA) according to the manufacturer’s protocol. Isolated RNA sample quality was assessed by High Sensitivity RNA Tapestation (Agilent Technologies, Santa Clara, CA, USA) and quantified by the Qubit 2.0 RNA HS assay (ThermoFisher, Waltham, MA, USA). Ribosomal RNA depletion was performed with the Ribo-Zero Plus rRNA Removal Kit (Illumina, San Diego, CA, USA). Samples were randomly primed and fragmented based on the manufacturer’s recommendation. The first strand was synthesized with Protoscript II Reverse Transcriptase with a longer extension period of approximately 40 min at 42 °C. All remaining steps for library construction were performed according to the NEBNext^®^ UltraTM II Directional RNA Library Prep Kit for Illumina^®^ (New England BioLabs Inc., Ipswich, MA, USA). The final quantity of the libraries was assessed by Qubit 2.0 (ThermoFisher), and quality was assessed by TapeStation D1000 ScreenTape (Agilent Technologies). Illumina^®^ 8-nt dual-indices were used. Equimolar pooling of libraries was performed based on QC values and sequenced on an Illumina^®^ [NovaSeq S4] (Illumina) with a read length configuration of 150 PE for 20 M PE reads per sample (10 M in each direction).

Raw data was initially processed using FastQC (v0.11.9) [28] and Trimmomatic (v0.40) [29] to identify and remove overrepresented artificial sequences such as adapters. Cleaned reads were further analyzed with Trimmomatic to remove low-quality bases. We used the “maxinfo” filtering in palindrome mode with the default parameters. Reads were subject to a quality threshold of 25, and if the retained length was less than 40 bp, they were removed from further analysis. The quality of sequencing data at the sample level before and after trimming and filtering was assessed using our in-house-developed method FQStat (v1.0) [30]. All samples yielded high-quality sequences and were carried on for downstream analysis. Transcript quantification was conducted using Salmon (v1.6.0) [31] based on EnsemblBacteria (Release 56) [32] assemblies for the two species. Transcripts per million (TPM) was used as the normalized abundance value for the transcripts, and DeSeq2 (v1.34.0) was used to assess differential expression [33]. A transcript was considered significantly differentially expressed if its fold change between the two conditions was above 2.0 and the adjusted Wald test *p*-value for this change using the Benjamini-Hochberg method was less than 0.05.

### 2.4. Metabolomics

Cell pellets were washed three times with cold PBS to remove any cell media left after collection. The cell pellets were then extracted using cold 100% methanol and spiked with 40 μL of 10 pinitol (the internal standard). A quality control (QC) sample was prepared by mixing the same amount of each sample into one. The supernatants were then dried down using a speed vacuum and then resuspended in 20 mg/mL methoxyamine hydrochloride reagent prepared in pure pyridine and incubated for 2 h at 37 °C on a platform shaker at 1000 rpm. Next, for derivatization, the MSTFA +1% TMCS derivatization (ThermoFisher) was added to each sample and incubated for 30 min at 37 °C on a platform shaker at 1000 rpm, followed by a centrifugation for 10 min at 16,000 g prior to transferring the mixture to GC vials for injection into GC-MS. The GC-MS analysis was carried out with an Agilent GC (Model 7890B) and MS Quadrupole (Model 5977A) (Agilent Technologies). The liquid injection was conducted using a PAL System RSI 85 (PAL, Lake Elmo, MN, USA). The injector temperature was 260 °C, and the MS transfer line was 230 °C. Metabolites were separated on a 5% phenyl 95% dimethylarylene siloxane HP-5MS 30 m, 0.25 mm, 0.25 μm capillary column (Agilent Technologies) at a constant flow rate of 1.5 mL·min^−1^ of helium as a carrier gas. One microliter of derivatized sample was injected into the injector operating in 1:5 split mode. The temperature of the column was initially set to 60 °C and increased at a rate of 10 °C·min^−1^ to 325 °C. The samples were run alongside the mixture of retention index C10–25 alkanes (DRO Mix Tennessee/Mississippi, Restek Corp., Bellefonte, PA, USA) for identification. The QC sample was injected three times in the sample sequence: once at the beginning, once after 10 samples, and one last time at the end to check on the instrument performance and adjust for instrument variation in the data processing. The samples were randomized for the sequence run.

The data was analyzed using MS-Dial (v4.9) for peak detection, deconvolution, alignment, quantification, normalization, and identification [34]. The putative identification of the metabolites was based on the Kovats retention index (RI) and the matching score of the mass spectra with the libraries. Two libraries were used: a local library made from running authentic standards with Kovats RI and a public spectrum library, the curated Kovats RI, with a total of 28,220 compounds (last edited 21 August 2022, which includes the Fiehn, RIKEN, and MoNA databases). The peaks were manually reviewed for peak shape, chromatogram alignment integrity, and MS/MS match, and the final list of compounds with RI similarities >95% was reported. The data was normalized based on the internal standard spiked in the samples during extraction and using LOWESS (locally weighted scatterplot smoothing) for QC-batch normalization.

Differential, functional, and pathway enrichment analyses have been performed using MetaboAnalyst 5.0 [35]. Metabolites with an adjusted *t*-test *p*-value < 0.05 using the Benjamini–Hochberg method were deemed significantly differentially expressed. Samples and metabolites were clustered using hierarchical clustering based on the Unweighted Pair Group Method with Arithmetic-mean (UPGMA) method [36] using MATLAB (v2022b, The MathWorks Inc., Natick, MA, USA). Metabolite signal values were normalized across samples to have zero-mean unit variance for each metabolite; average linkage was employed in UPGMA; and Pearson’s correlation was used as the distance measure between metabolites.

### 2.5. Proteomics

Cell pellets washed with cold PBS were further washed once with 70% ethanol and air-dried. The proteins were solubilized in 400 μL of 7 M urea, 2 M thiourea, 5 mM DTT, 0.1 M Tris, pH 8, and reduced for 2 h at 37 °C. Protein amounts were assayed using the CBX kit (G-Bioscience, St. Louis, MO, USA), and an aliquot of each sample was alkylated with iodoacetamide. Lys-C digestion was carried out at a 1:20 *w*/*w* ratio for 4 h in 4 M urea before dilution to 1 M urea and digestion with trypsin (1:20 ratio) for 20 h at 37 °C. 5 μg of each digest was diluted to 45 μL with 0.5% TFA and 5 μL of 100 fmol/μL of phosphorylase B (MassPREP, Waters Corp., Milford, MA, USA) added. 0.5 μg (5 μL) of each sample was analyzed, containing 50 fmol of phosphorylase B. Each digest was run by a Thermo Ultimate 3000 nanoLC-MS/MS using a 2 h gradient on a Waters CSH 0.075 mm × 250 mm C18 column feeding into a Thermo Orbitrap Eclipse mass spectrometer. The order of sample runs was randomized.

All MS/MS samples were analyzed using Mascot (Matrix Science, London, UK; v2.7.0). Mascot was set to search the cRAP database from the global proteome machine (v1.0), the JCM5818 (2027 sequences) and JCM6985 (1831 sequences) protein databases obtained from the NCBI RefSeq collection (release 206), and the phosphorylase B assuming the digestion enzyme trypsin with a fragment ion mass tolerance of 0.6 Da and a parent ion tolerance of 10.0 PPM. Deamidated asparagine and glutamine and oxidation of methionine were specified in Mascot as variable modifications. The carbamidomethyl of cysteine was specified in Mascot as a fixed modification. The quantitation of the proteins was conducted using Proteome Discoverer (ThermoFisher; v2.4). The data were searched using a decoy database to set the false discovery rate to 1% (high confidence). Only proteins with a minimum of 2 peptides and 5 peptide spectrum matches (PSMs) were reported. The peptides were quantified using the precursor abundance based on intensity, normalized based on the specified protein, and raw abundances are reported. The protein ratios are calculated using the summed abundance for each replicate separately. The significance of differential expression was assessed using a *t*-test with adjusted *p*-values based on the Benjamini–Hochberg method following LOWESS normalization.

### 2.6. Meta-Multi-Omics Network Construction

**Figure 1:**
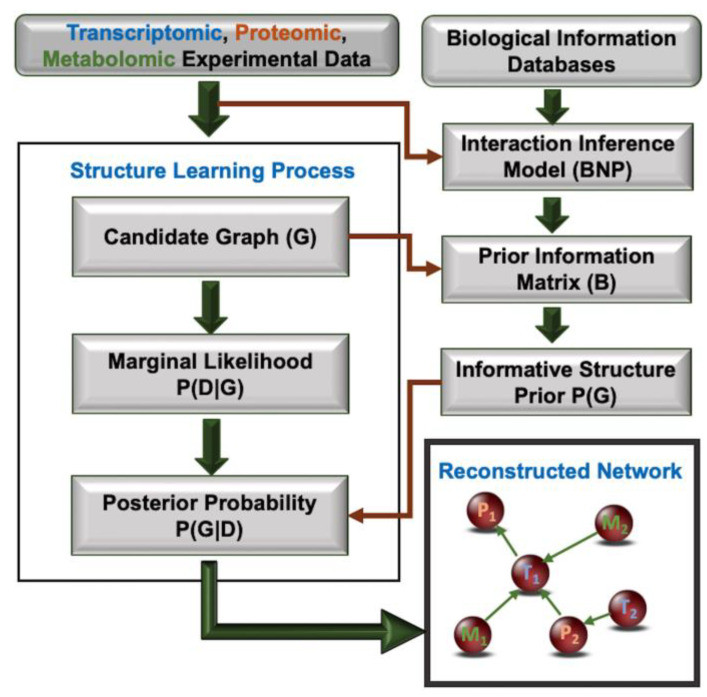
Workflow for network generation using meta-multi-omics data with external knowledge. G—Graph; D—Data; BNP—Bayesian Network Prior construct; B—molecular interaction probability matrix based on BNP; Pi, Mi, and Ti—ith input protein, metabolite, and transcript, respectively.

The overall workflow of the proposed method for building multi-omics interaction networks is depicted in Figure 1. The interaction inference model, referred to as the Bayesian network prior (BNP) model, is used to infer the interaction probability of two molecules based on external knowledge and given experimental data. Various experimental techniques may imply an interaction between two molecules. However, there remains stochastic uncertainty regarding the reliability of a true interaction. BNP represents the dependency structure between “different experimental evidence types that imply molecular interaction” and the “event, molecular interaction” [18,37]. Therefore, BNP is itself a BN, with one node representing “molecular interaction” and the remaining nodes representing “different evidence types”.

The topology of BNP was learned using the observation matrix, which consists of pairs of molecules as rows and evidence types attesting to their interaction as columns. We use the term “interaction” in its broadest form, which can represent regulation or co-regulation for transcripts, protein-protein interactions for proteins, metabolic reaction involvement for an enzyme-metabolite pair, etc. If two molecules were deemed interacting/associated in an external database, we included that pair in our evidence matrix, which is used to build BNP. This matrix was generated by automatically extracting the interaction information from sources such as Gene Expression Omnibus (GEO) for co-expression [38], KEGG (Release 104) [39], NCI/Nature Pathway Interaction Database (PID; v2.0) [40], Reactome (v82) [41], Integrated Interactome System (IIS; v1.0.2) [42], and Biological General Repository for Interaction Datasets (BioGRID; v4.4.214) [43]. If the interaction information obtained from a database did not include the organisms for which the meta-multi-omics data came from, such interactions were inferred by orthology. For each row, if an evidence type, e.g., a yeast-two-hybrid, co-purification, or co-expression experiment, implies the interaction of the pair, we placed a 1 (and otherwise a 0) in the matrix. A “molecular interaction” column was added, and it was called 1 if two or more evidence types implied the interaction of a molecule pair along a row.

Based on this evidence matrix, BNP was learned using the Bayesian information criterion (BIC) score [44] and the hill climbing method [45] with the bnlearn (v4.6.1) R package [46]. The strength of the probabilistic relations expressed by the edges of the BNP was measured using Friedman’s bootstrap method with 1000 repeats [47]. Model averaging was used to build a consensus network containing only the edges with significant strength values [48].

Given *n* molecules across *k* samples from two groups, e.g., case and control, an *n × k* data matrix was formed. This data matrix contained appended signal values from different omics types along its rows. Data preprocessing for BN structure learning was conducted as previously described [18,49,50]. Briefly, for each molecule, fold change values between each possible pair of samples in the two groups were obtained. Let *N1* be the number of samples in the first group and *N2* be the number of samples in the second group. For each molecule, we calculated *N1 × N2* fold change values: the first sample in the second group was compared with the first sample in the first group, the first sample in the second group was compared with the second sample in the first group, etc. This “observation matrix”, which contained *n* columns for the molecules that constitute the nodes in the interaction network and *N1 × N2* rows for the fold change values, was used as the input experimental data for the structure learning process. MEMINEX currently handles experimental data that stems from two groups of samples, e.g., case-control. Experiments that involve multiple groups can either be analyzed by considering two groups at a time, or by identifying a “combined” fold change value for each molecule across different groups of samples.

BNP was instantiated for each pair of molecules with both the given experimental data and existing external evidence. This resulted in a probability value for the BNP’s “molecular interaction” node for the pair, incorporating external knowledge already embedded in the BNP. This process is repeated for every pair in the *n* molecules, resulting in an *n × n* “probability of interaction” matrix, *B*, which constitutes the degree of belief that two molecules interact based on external knowledge and experimental data. For a candidate graph *G* in the BN structure learning phase, *P*(*G*) is calculated using *B* as previously described [18].

A BN structure learning algorithm should find the graph *G* that maximizes the “maximum *a posteriori*” (MAP) measure, *P*(*G*|*D*), where *D* represents the input data. However, using the equality *P*(*G*|*D*) = [*P*(*D*|*G*) *P*(*G*)]/*P*(*D*), existing methods find *G* that maximizes *P*(*D*|*G*), the “maximum likelihood” (ML) parameter [45]. This is justified because *P*(*D*) is the same for all *G* (as *D* is observed) and there is no model to calculate *P*(*G*) for each candidate graph. *P*(*D*|*G*) can easily be calculated using methods such as Bayesian Dirichlet equivalent (BDe) scoring [51]. As the proposed method enables us to calculate *P*(*G*) for a candidate graph *G* during the search process of the BN structure learning algorithm, we can maximize the optimum MAP measure instead of the suboptimal ML parameter [52,53,54]. We previously showed in human single omics data that the proposed workflow outperformed existing network building approaches in identifying pathways active in a phenotype and in building interaction networks with a 15–40% improvement in overall performance [18,49,50,55].

## 3. Results

The growth of *L. kefiranofaciens* was higher at 37 °C than at 30 °C. However, *L. kefiranofaciens* did not grow as well at 37 °C in the monoculture compared to the co-culture. On the other hand, the growth of *L. kefiri* was higher at 30 °C than at 37 °C; the co-culture grew better at both temperatures than the monocultures.

### 3.1. Transcriptomics

We quantified 2587 *L. kefiri* and 2368 *L. kefiranofaciens* genes. 2051 *L. kefiri* genes were significantly downregulated at 37 °C compared to 30 °C, while only 4 *L. kefiri* genes were significantly upregulated at 37 °C compared to 30 °C. Conversely, we found 1620 *L. kefiranofaciens* genes significantly upregulated at 37 °C compared to 30 °C, while only 4 *L. kefiranofaciens* genes were significantly downregulated at 37 °C compared to 30 °C. In Table 1, we show the top 20 up- and downregulated genes at 37 °C compared to 30 °C. The complete list of significantly differentially expressed genes can be found in Supplemental File S1.

### 3.2. Metabolomics

**Figure 2:**
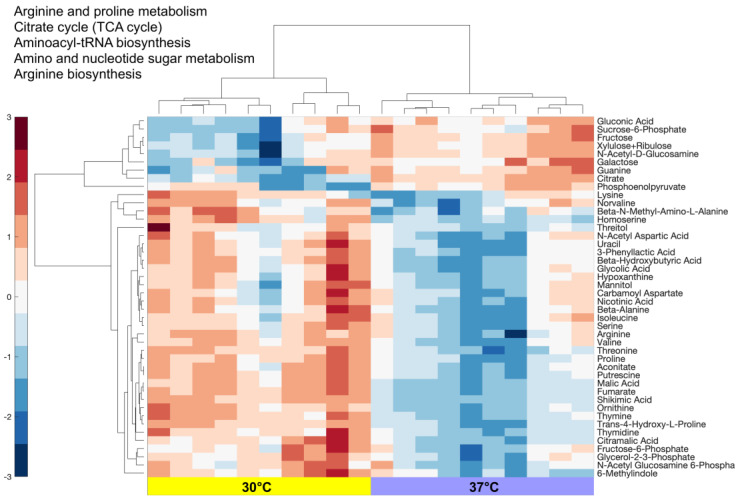
Hierarchical clustering of samples/metabolites and pathways enriched with the 44 differentially expressed metabolites. The scale on the left shows the range of the standardized row expression levels.

We analyzed the normalized data with MetaboAnalyst 5.0 [35] and identified 44 significantly differentially expressed (Benjamini–Hochberg-corrected *p*-value < 0.05) metabolites (Figure 2, Supplemental File S1) out of a total of 100 measured. These metabolites were mostly downregulated at 37 °C (35/44). Pathways significantly enriched (adjusted *p*-value < 0.05) by the 44 metabolites are shown in Figure 2. These pathways indicate the biological mechanisms that were potentially active from 30 °C to 37 °C.

### 3.3. Proteomics

**Figure 3:**
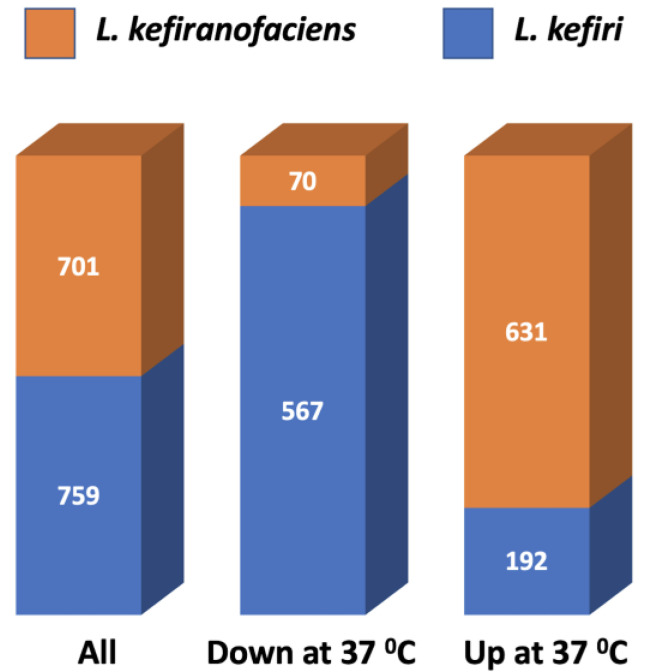
Breakdown of significantly differentially expressed proteins by organism and by directionality.

Based on an adjusted *p*-value cut-off of 0.05, there were 1460 significantly differentially expressed proteins between 37 °C and 30 °C (Figure 3, Supplemental File S1). These results verified those of the transcriptomic data: most of the proteins upregulated at 37 °C were from *L. kefiranofaciens*, and most of the proteins downregulated at 37 °C were from *L. kefiri*. When we further constrained this list with a fold change of 2.0, the same observation held: 239/259 proteins upregulated at 37 °C were from *L. kefiranofaciens*, and 558/650 proteins downregulated at 37 °C were from *L. kefiri*.

### 3.4. Combined Multi-Omics Analysis

Using interaction information gathered from external databases, we learned the BNP structure, which represents the dependency between different evidence types that imply interaction and the event “molecular interaction.” The reliability of the BNP construct was assessed using 5-fold cross-validation. We left 20% of the pairs in the evidence matrix out and built the BNP structure as described using the remaining 80% of the rows. Then, using this model, we predicted the “molecular interaction” event for the left-out 20% pairs using BN inference and assessed if it was correctly called. We achieved an area under the curve (AUC) of 92%, implying high accuracy in predicting interactions.

Following the workflow described in Figure 1, we obtained an interaction network for the synthetic bacterial consortium using transcriptomic, proteomic, and metabolomic data. We ranked the statistically significant differentially expressed up- and down-regulated molecules separately for each omics type based on absolute fold change. We then picked the top 10 molecules from these lists, accounting for 20 molecules per omics for a total of 60 molecules. Our choice was motivated by obtaining a balanced representation of each omics type without overwhelming visual inspection of the interaction network and run times. There were only 9 significantly upregulated metabolites, so for metabolomics, we picked the top 9 upregulated and top 11 downregulated molecules for a total of 20. The molecules used in multi-omics analysis are highlighted in Supplemental File S1. Following data preprocessing as previously described [18,49], an interaction network was learned using the max-min hill climbing (*mmhc*) algorithm in *bnlearn*. This structure learning was conducted with the incorporation of external knowledge through the BNP construct, which led to the calculation of *P*(*G*) for candidate graphs and consequently resulted in the optimization of the MAP measure in the search process.

As detailed in Figure 1, during the structure learning phase, for each candidate graph, *G*, the following steps were performed. For each pair of 60 molecules, we instantiated BNP to infer the interaction probability for the pair based on the observed experimental data and external knowledge, which is modeled in BNP. This resulted in a *B* matrix that represented the probability of interaction for every pair of molecules. Using our previously described method, we calculated *P*(*G*) using the topology of *G* and the interaction probability matrix *B* [18,55]. *P*(*G*) was incorporated in the *mmhc* learning algorithm, so *P*(*G*|*D*), the maximum *a posteriori* measure, is maximized during the search process instead of *P*(*D*|*G*), the maximum likelihood parameter, which is the default optimization parameter for the algorithm. At the end of the structure learning process, an interaction graph for the 60 molecules was found.

**Figure 4:**
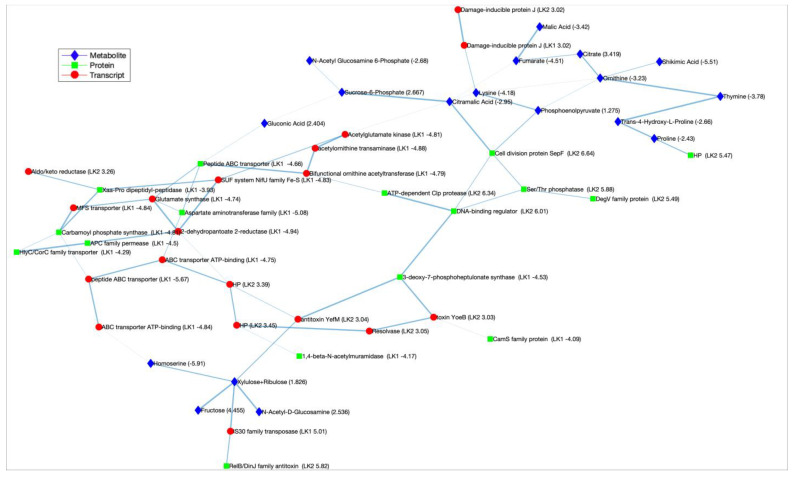
Multi-omics interaction network. Fold change values (37 °C/30 °C) are shown in parentheses. LK1—*L. kefiri*; LK2—*L. kefiranofaciens*. Edge thickness denotes confidence in the interaction.

We repeated this process 1000 times on bootstrapped data sets and obtained a “link strength” value between every pair of molecules. This value, which is between 0 and 1, represents the fraction of times the final learned network had a link between the pair out of the 1000 runs. For example, if a nucleotide pair had a strength value of 0.9, this meant that in 900 out of the 1000 learned networks, the final interaction graph had a link between the pair. An optimum cut-off for strength values was calculated [48], and links with a strength value below the cut-off were removed, retaining only the edges with significant strengths. The total run time was 4.37 min on a Linux machine with 128 GB of RAM and a dual Intel^®^ Xeon^®^ E7-8870 CPU with 16 cores. The final network is shown in Figure 4, where the link thickness is proportional to the strength value between the pair of molecules. Nodes represent the different molecule types (metabolites, proteins, and transcripts) and are color-coded. The final network indicates that the proposed method can obtain interactions between multiple omics types, which would not be possible with single-omics analysis.

### 3.5. Comparison with Other Methods

The 60 molecules used in our analysis were also used as input to OmicsNet [4], a multiomics integrator using external knowledge and an established workflow implementing correlation-based network generation [10]. OmicsNet identified the direct interacting partners of our input list from molecular interaction databases, expanding the final network with nodes that are not the input molecules. We sifted edges that exist between input molecules to compare with our results and identified four such links between (i) 3-deoxy-7-phosphoheptulonate synthase (protein)—Phosphoenolpyruvate (metabolite), (ii) damage-inducible protein J (transcript)—lysine (metabolite), (iii) IS30 family transposase (transcript)—xylulose, and (iv) 2-dehydropantoate 2-reductase (transcript)—proline (metabolite). All but the last, i.e., three out of four interactions were also identified by our approach.

As shown in Figure 4, MEMINEX identified 69 edges with significant strength among the 60 input molecules. To obtain a network of similar size, we used a *p*-value cut-off of 10^−6^ in the correlation-based approach to define an edge between two molecules. This resulted in a network with 95 edges, as shown in Supplementary Figure S2. The edges of the two networks are compared in detail in Supplementary File S2. The two networks shared 28 edges, which represented a significant overlap (BH-corrected *p*-value < 3 × 10^−9^) [56]. When we investigated edges that establish an interaction between different omics types, we found 25 out of 69 (or ~37%) of our network’s edges and 15 out of 95 (or ~16%) of the correlation-based network’s edges to be of such nature.

## 4. Discussion

In this paper, we present an algorithm that identifies interactions between different types of molecules, e.g., proteins, transcripts, and metabolites, in a bacterial community. The proposed approach uses a Bayesian network (BN) framework that incorporates external knowledge when identifying the final network of interactions. We optimize the search process during the BN structure learning phase, which is different from standard network-building approaches. As a model synthetic consortium, we chose two of the most prominent members of kefir grains, *L. kefiri* and *L. kefiranofaciens*. Our individual omics analysis indicated that going from 30 °C to 37 °C, there was an increase in cellular activity in *L. kefiranofaciens*, while the opposite was true for *L. kefiri*. Interestingly, *L. kefiranofaciens* did not show growth at 37 °C in the monoculture, but in the co-culture, not only did it show growth at both temperatures, but it also showed significant upregulation in its molecular machinery going from 30 °C to 37 °C.

Transcriptomics results, available in Supplementary Table S1, show that most of the genes upregulated at 37 °C belonged to *L. kefiranofaciens* and most of the genes downregulated at 37 °C belonged to *L. kefiri*. Out of the top ten upregulated genes at 37 °C shown in Table 1, all belonged to *L. kefiranofaciens* except for one gene. The only upregulated *L. kefiri* gene, which also had the highest fold change, was the IS30 family transposase. IS30 transposase elements have been shown to be associated with environmental adaptation and stress resistance, specifically being more upregulated for heat stress [57]. The other nine upregulated genes that belong to *L. kefiranofaciens* were hypothetical proteins, the toxin/antotoxin (TA) system, aldo/keto reductase, resolvase, and DNA damage-inducible protein J. These genes play a role in survival at high temperatures and contribute to the maintenance of plasmid stability in *L. kefiranofaciens*. There were no *L. kefiranofaciens* genes among the top ten downregulated genes at 37 °C. The top ten downregulated genes that belong to *L. kefiri* were involved in transporting peptides and sugar inside the cells. Their downregulation potentially implies the abolition of growth.

Based on the metabolomics analysis shown in Figure 2, pathways downregulated by the co-culture at 37 °C were metabolism and biosynthesis of arginine and proline, metabolism of amino sugar and nucleotide sugar, and biosynthesis of aminoacyl-tRNA. The upregulated metabolites were significantly associated with the glycolysis and carbohydrate metabolism pathways. These results help explain the metabolic mechanisms at play during the increased activity of *L. kefiranofaciens* and the stalled growth of *L. kefiri* at 37 °C.

Our proteomics results were in line with those of transcriptomics, both from quantitative and functional perspectives. Most of the proteins that showed increased abundance at 37 °C belonged to *L. kefiranofaciens*, while most of the proteins with decreased abundance at 37 °C were from *L. kefiri*. Toxin/antitoxin system proteins, carbohydrate metabolism, fatty acid metabolism, and membrane proteins were among the top upregulated proteins, while proteins involved in peptide and carbohydrate metabolism in *L. kefiri* were downregulated. These proteomics results supported the inverse activity of *L. kefiranofaciens* and *L. kefiri* at 37 °C.

Our individual omics analysis showed promising results, explaining putative biological mechanisms that can be inferred through a single-omics type. However, each of the single-omics results explains only one facet of the underlying functionality. A combined multi-omics analysis of the three different datasets holds promise for presenting a holistic picture of the biological mechanisms underlying the differential growth of *L. kefiri* and *L. kefiranofaciens* at 30 °C and 37 °C, respectively. Using the top 20 transcripts, metabolites, and proteins that resulted from our individual omics analysis and following the workflow described in Figure 1, we obtained the meta-multi-omics interaction network shown in Figure 4. As shown in this figure, we were able to integrate diverse omics data types and obtain connections between different molecule classes, inferring multi-scale interactions.

Our results suggested that two toxin/antitoxin (TA) systems were active at 37 °C: the YefM/YoeB system at the transcript level and the RelB/DinJ system at the protein level. The YefM/YoeB system was associated with two downregulated *L. kefiri* proteins, 3-deoxy-7-phoshoheptulonate synthase and CamS, and two upregulated *L. kefiranofaciens* transcripts coding for resolvase and a hypothetical protein. The RelB/DinJ TA system of *L. kefiranofaciens* was associated with the IS30 family transposase of *L. kefiri*. These two systems were also associated with several metabolites, primarily carbohydrates, such as xylulose, ribulose, fructose, and homoserine.

The bacterial type II TA systems are distributed in bacterial chromosomes and mobile genetic elements, such as plasmids, that are associated with various transposons and resolvases. Activation of the TA system can modulate bacterial adaptation to different stress conditions. This process enables bacteria to overcome adverse environmental stress and plays a key role in the maintenance of genetic materials, biofilm formation, phage inhibition, and persister formation [58,59]. The CamS family proteins participate in the binding of cells with other cells or with the extracellular matrix to begin aggregation and promote biofilm formation. This is critical for probiotic bacteria such as Lactobacillus strains to increase their resistance to temperature, low pH, and mechanical stress [60].

An upregulated *L. kefiranofaciens* DNA-binding regulator protein was found to be associated with the downregulated *L. kefiri* 3-deoxy-7-phoshoheptulonate synthase protein. This protein is the first enzyme in the shikimate pathway responsible for the biosynthesis of aromatic amino acids, such as phenylalanine, tyrosine, and tryptophan; ATP-dependent Clp protease; DegV family proteins; the cell division protein Sep(F); Ser/Thr phosphatase; and some metabolites, such as citramalic acid and phosphoenolpyruvate (PEP). The ATP-dependent Clp protease in bacteria is responsible for the degradation of the antitoxin part of the TA system so that the toxin is free to inhibit several cell components [61]. The DegV family of proteins is essential for the activation of exogenous fatty acids so they can be incorporated into phospholipids.

Based on the meta-multi-omics interaction network, the DNA-binding regulator of *L. kefiranofaciens* might help control the synthesis of these proteins in the cell. Our individual omics results showed that *L. kefiranofaciens* was predominant in the mixed culture at 37 °C but not at 30 °C. The carbohydrate and protein/peptide transporters from the major facilitator superfamily (MFS) and ATP-binding cassette (ABC) transporters of *L. kefiri* were down-regulated, supporting these results. Our results underscore the utility of an approach that integrates multifarious omics data for a bacterial community using both experimental results and external knowledge, optimizing the network construction step.

Compared to Omicsnet, which uses external knowledge to identify multi-omics interactions for input molecules, MEMINEX established a wider range of literature-based interaction information used in its BNP construct. Furthermore, our method incorporated this information into its model to determine the final network topology, unlike Omicsnet, which establishes links using a hard cut-off based on their existence in external databases. MEMINEX incorporates experimental data with the modeled external knowledge and fuses these two information sources to calculate the final interaction network (Figure 1). The proposed approach identified three out of four interactions found by Omicsnet that involved the input molecules, showing high concordance.

Our calculated network (Figure 4) and the network identified using a correlation-based approach (Supplementary Figure S1) were more comparable than the Omicsnet network and showed high overlap among their identified edges. However, the correlation network’s edges were predominantly between the same omics types, while MEMINEX identified a much larger set of edges between different omics types: about 36% of MMEINEX edges were between different omics types, while this percentage was only ~16% for the correlation network (Supplementary File S2). From a functional perspective, the correlation network also identified the TA system activation as seen in the MEMINEX network.

Our comparison results provided more reliability for the identified interactions, as there were significant overlaps with the results of other network-generation software. These results also showed that the proposed approach establishes an adequate amount of multi-omics edges (interactions between different omics types), casts a wider net for external interaction knowledge retrieval, and provides a novel framework that fuses modeled external knowledge with experimental data to determine the final network topology.

Despite successful multi-omics data generation for a bacterial community and the establishment of an algorithm that can calculate a meta-multi-omics interaction network for such data, our study suffers from several limitations. First, different synthetic consortia can be tested with the proposed algorithm to assess the generalizability of our results. Second, instead of a synthetic consortium, kefir grains themselves can be used as the biological sample to infer multi-omics interaction dynamics. Third, the proposed algorithm can be further developed to be applied to time-series data, so community dynamics and their implications for the underlying multi-omics interactions are explored. We believe these limitations can be overcome with future studies, and our current work is nevertheless an important milestone in establishing a prototype for our algorithm with a viable testbed bacterial community that demonstrates meta-multi-omics interaction networks.

## 5. Conclusions

Understanding the molecular interactions in a microbial community is a challenging task, as there are complex activities among the members of the community that involve different macromolecules. A step in the right direction is the multi-omics approach, which can reliably be generated using current technologies. However, the analysis of such meta-multi-omics data is the bottleneck due to its high dimensionality and variability. We tackle this problem using probabilistic graph representations. We believe network-based approaches present a common ground for both computational and life scientists that is intuitive and easy to interpret. Furthermore, we advocate that computational tools designed for biological or clinical data analysis should not solely rely on experimental outcomes but also make use of existing scientific knowledge.

We present an approach based on these two main pillars, generating interaction networks for meta-multi-omics data using stochastic modeling and external knowledge. We present an accompanying website that is freely available to the academic community to use the software that implements the proposed approach. The generated interaction networks can be used for mechanistic modeling and hypothesis generation for further testing. Using the proposed approach, existing meta-multi-omics data can be re-analyzed, mining additional information from experimental data that has taken significant resources to obtain.

## Figures and Tables

**Table 1:** Top 20 significantly up/downregulated genes between 37 °C and 30 °C. LK1—*L. kefiri*; LK2—*L. kefiranofaciens*; FC—Fold Change; p_adj—adjusted *p*-value.

Gene ID/Organism	log2 FC	p_adj	Gene Description
12225/LK1	5.01	3.64 × 10^−02^	IS30 family transposase
02047/LK2	3.45	6.80 × 10^−114^	hypothetical protein
01570/LK2	3.39	4.03 × 10^−133^	hypothetical protein
01848/LK2	3.26	2.12 × 10^−245^	Aldo/keto reductase
02423/LK2	3.22	2.52 × 10^−146^	hypothetical protein
02046/LK2	3.05	8.80 × 10^−82^	Resolvase, N terminal domain
01913/LK2	3.04	7.70 × 10^−112^	antitoxin YefM
01912/LK2	3.03	4.81 × 10^−67^	toxin YoeB
02422/LK2	3.02	5.27 × 10^−148^	DNA-damage-inducible protein J
12115/LK1	3.02	5.56 × 10^−148^	damage-inducible protein J
03195/LK1	−5.68	2.39 × 10^−285^	peptide ABC transporter substrate-binding protein
10965/LK1	−4.94	2.38 × 10^−72^	2-dehydropantoate 2-reductase
07015/LK1	−4.88	6.54 × 10^−222^	acetylornithine transaminase
05460/LK1	−4.85	1.88 × 10^−258^	MFS transporter
05315/LK1	−4.85	4.06 × 10^−162^	ABC transporter ATP-binding protein
01085/LK1	−4.83	9.68 × 10^−78^	SUF system NifU family Fe-S cluster assembly protein
07020/LK1	−4.81	1.90 × 10^−145^	acetylglutamate kinase
07025/LK1	−4.79	3.71 × 10^−172^	bifunctional ornithine acetyltransferase/N-acetylglutamate synthase
05320/LK1	−4.75	5.02 × 10^−156^	ABC transporter ATP-binding protein
01060/LK1	−4.75	3.16 × 10^−164^	glutamate synthase

## Data Availability

RNA-seq data is deposited in the NCBI GEO database with the accession number GSE229515. The mass spectrometry proteomics data is deposited to the ProteomeXchange Consortium via the PRIDE [62] partner repository with the dataset identifier PXD042954. The metabolomics data is available at the NIH Common Fund’s National Metabolomics Data Repository (NMDR) website, the Metabolomics Workbench [63], where it has been assigned Study ID ST002741. The software used in this publication is available at http://otulab.unl.edu/MEMINEX (accessed on 1 August 2023).

## Supplementary Materials

The following supporting information can be downloaded at: https://www.mdpi.com/article/10.3390/cells12151998/s1. File S1: List of significantly differentially expressed transcripts, metabolites, and proteins. File S2: Detailed comparison of MEMINEX and correlation networks listing all, overlapping, and multi-omics edges. Figure S1: Growth curves for mono- and co-cultures at 30 °C (A) and 37 °C (B). LK1—*L. kefiri*; LK2—*L. kefiranofaciens*; Mixed—Co-culture. Figure S2: Multi-omics interaction network based on a correlation-based approach.
