# Supplementary material for: Integration of Meta-Multi-Omics Data Using Probabilistic Graphs and External Knowledge
Source: Cells. 2023 Aug 4;12(15):1998. doi: 10.3390/cells12151998 (PMC10417344; doi:10.3390/cells12151998)
Supplement: Supplementary file 1 [file cells-12-01998-s001.zip › Supplementary_Figures.docx]

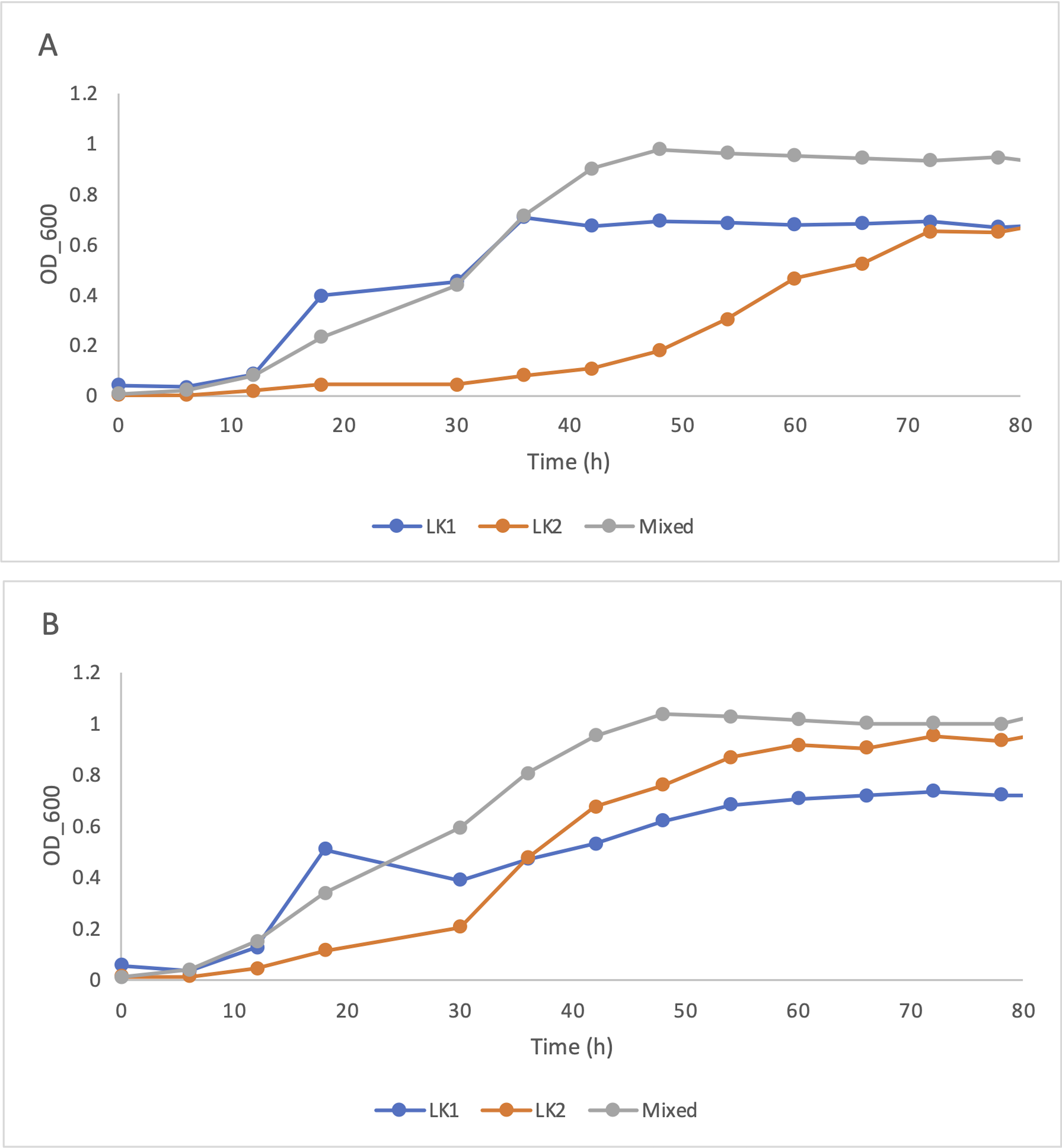


Figure S1: Growth curves for mono- and co-cultures at 30 °C (A) and 37 °C (B). LK1—*L. kefiri*; LK2—*L. kefiranofaciens*; Mixed—Co-culture





Figure S2: Multi-omics interaction network based on a correlation-based approach.
